# Asymmetry of inverted-topology repeats in the AE1 anion exchanger suggests an elevator-like mechanism

**DOI:** 10.1085/jgp.201711836

**Published:** 2017-12-04

**Authors:** Emel Ficici, José D. Faraldo-Gómez, Michael L. Jennings, Lucy R. Forrest

**Affiliations:** 1 Theoretical Molecular Biophysics Laboratory, National Heart, Lung and Blood Institute, National Institutes of Health, Bethesda, MD; 2 Computational Structural Biology Unit, National Institute of Neurological Disorders and Stroke, National Institutes of Health, Bethesda, MD; 3 Department of Physiology and Biophysics, University of Arkansas for Medical Sciences, Little Rock, AR

## Abstract

The membrane transporter anion exchanger 1 (AE1), or band 3, is a key component in the processes of carbon-dioxide transport in the blood and urinary acidification in the renal collecting duct. In both erythrocytes and the basolateral membrane of the collecting-duct α-intercalated cells, the role of AE1 is to catalyze a one-for-one exchange of chloride for bicarbonate. After decades of biochemical and functional studies, the structure of the transmembrane region of AE1, which catalyzes the anion-exchange reaction, has finally been determined. Each protomer of the AE1 dimer comprises two repeats with inverted transmembrane topologies, but the structures of these repeats differ. This asymmetry causes the putative substrate-binding site to be exposed only to the extracellular space, consistent with the expectation that anion exchange occurs via an alternating-access mechanism. Here, we hypothesize that the unknown, inward-facing conformation results from inversion of this asymmetry, and we propose a model of this state constructed using repeat-swap homology modeling. By comparing this inward-facing model with the outward-facing experimental structure, we predict that the mechanism of AE1 involves an elevator-like motion of the substrate-binding domain relative to the nearly stationary dimerization domain and to the membrane plane. This hypothesis is in qualitative agreement with a wide range of biochemical and functional data, which we review in detail, and suggests new avenues of experimentation.

## Abstract (precis)

Anion exchanger 1 catalyzes the transmembrane antiport of chloride and bicarbonate ions through a mechanism that has remained unclear. By modeling its inward-facing state and comparing it with the known outward-facing form, Ficici et al. hypothesize that this transporter features an elevator-like mechanism.

## Introduction

The major integral membrane protein of the human erythrocyte, known as band 3 or anion exchanger 1 (AE1), catalyzes the electroneutral exchange of Cl^−^ and HCO_3_^−^ across the plasma membrane, which is one of the key steps in CO_2_ transport in the blood (Wieth et al., 1982; Passow, 1986). The *AE1* gene is expressed not only in erythroid cells but also in the kidney (Brosius et al., 1989), where it acts as a base exit pathway in acid-secreting α-intercalated cells of the collecting tubule (Schuster et al., 1986; Brosius et al., 1989). AE1 is a member of the SLC4 family of transporters, which includes Cl^−^/HCO_3_^−^ exchangers as well as electrogenic and electroneutral Na^+^/HCO_3_^−^ symporters (Romero et al., 2013). In addition to monovalent anions, AE1 can also transport SO_4_^2−^ under low-pH conditions (Gunn, 1972; Ku et al., 1979; Passow, 1986).

AE1 is an ∼95-kD glycoprotein consisting of two major domains with distinct functions (Steck, 1978). The N-terminal 360 residues form a water-soluble cytoplasmic domain that serves as an anchoring site for the membrane skeleton (Bennett, 1985; Low, 1986; Zhang et al., 2000). The C-terminal domain (∼530 residues) resides within the membrane and forms tightly associated dimers (Reithmeier, 1979), which catalyze the anion-exchange reaction. Functional studies using the stilbenedisulfonate inhibitor 4,4′-diisothiocyanatodihydrostilbene-2-2′-disulfonate (H_2_DIDS; Cabantchik and Rothstein, 1974), which binds to each of the AE1 protomers (Jennings and Passow, 1979), suggested that the mechanisms of the protomers are independent of one another (Lepke et al., 1976; Ship et al., 1977; Wieth, 1979).

**Figure 1.**
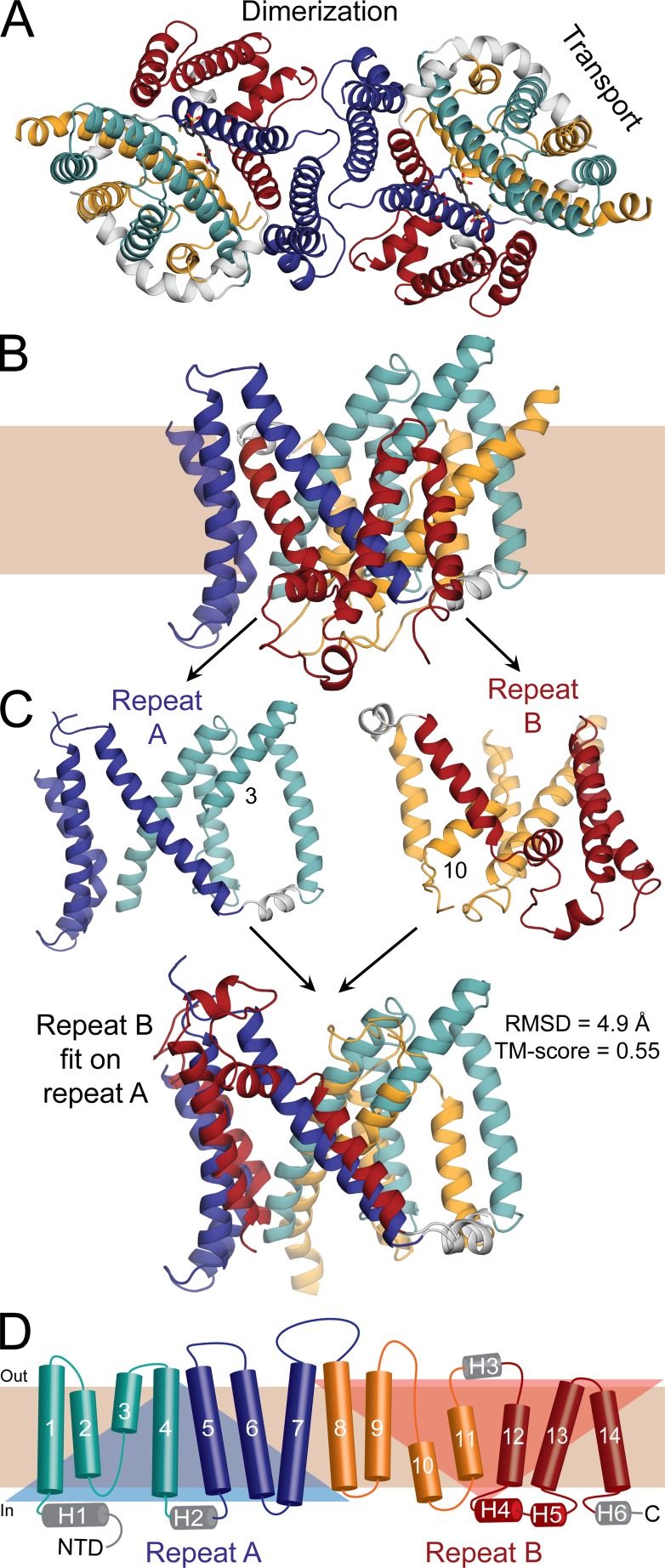
**Structure of the C-terminal domain of human erythrocyte AE1 in an outward-open conformation. (A and B)** View of the structure (PDB ID 4YZF) shown as cartoon helices from the extracellular side as a dimer (A) or along the plane of the membrane as a protomer (B). Helices in the transport domain are colored cyan and orange, whereas helices in the dimerization domain are colored red and blue. Peripheral helices are colored gray. **(C)** Structural repeats A and B, consisting of transmembrane (TM) helices 1–7 (residues 381–635) and TMs 8–14 (residues 636–880), respectively, are shown alone (top) or with repeat B superposed onto repeat A, using TMalign (bottom). The RMSD and TM score resulting from this superposition show that the repeats share the same architecture but display clear differences. The alignment matches 195 residues out of 226. The TM segments that are not continuous helices, TM3 and TM10, are labeled. **(D)** Transmembrane topology of AE1 highlighting the structural repeats (transparent triangles), and colored according to A. Helices are indicated with cylinders and the extended chain in TM helices 3 and 10 is shown as arrows. Helices outside of the membrane are labeled H1–H6. NTD, N-terminal domain.

Recently, a crystal structure of the dimeric membrane domain of human AE1 was determined at a resolution of 3.5 Å (Arakawa et al., 2015), providing a crucial stepping stone for new mechanistic studies. In each protomer, the membrane domain consists of 14 transmembrane (TM) segments, which appear to be arranged into two distinct subdomains comprising (1) TM1–TM4 and TM8–TM11 and (2) TM5–TM7 and TM12–TM14 (Arakawa et al., 2015), which we will refer to as “transport” and “dimerization” domains, respectively (Fig. 1). The overall architecture of AE1, which is likely to be shared by other proteins in the SLC4 family, is similar to that observed for two other transporter families (Chang and Geertsma, 2017): the SLC23 family, represented by the uracil and xanthine transporters UraA (Lu et al., 2011; Yu et al., 2017) and UapA (Alguel et al., 2016); and the SLC26 or SulP family, represented by a fumarate transporter known as SLC26Dg (Geertsma et al., 2015). A striking feature of this architecture is that within the transport domain, TM3 and TM10 are α-helical only partway across the membrane; the remaining residues adopt an extended conformation, which enables the N termini of these two short helices to approach each other roughly in the middle of the membrane. The anion-binding site in AE1 (i.e., the site occupied alternatively by Cl^−^ or HCO_3_^−^) is likely to be precisely between the ends of TM3 and TM10 (Arakawa et al., 2015). No anions were detected in the AE1 crystal structure, most likely because the protein is inhibited by H_2_DIDS. Nevertheless, the putative location of this anion-binding site would be consistent with what is seen in the structures of UraA and UapA (Lu et al., 2011; Alguel et al., 2016; Yu et al., 2017); it would also be in keeping with the general observation that anion-binding sites in proteins are often at the N-terminal (positive) ends of α-helices, where the anion interacts with exposed amino groups from the protein backbone and with the electrostatic dipole of the nearest helical turns (Hol et al., 1978; Faraldo-Gómez and Roux, 2004; Sengupta et al., 2005).

AE1 is believed to operate with an alternating-access mechanism (Jardetzky, 1966); i.e., the protein is expected to interconvert between two major conformational states that expose the anion-binding site to either the extracellular or the intracellular space, but not both simultaneously (Gunn and Fröhlich, 1979; Furuya et al., 1984; Passow, 1986). As mentioned previously, the crystallized AE1 protein reacts with H_2_DIDS (Arakawa et al., 2015), which concurrently cross-links to two lysine residues (K539 and K851) in each protomer (Jennings and Passow, 1979; Okubo et al., 1994), preferentially in an outward-facing (OF) state (Fröhlich, 1982; Jennings, 1982; Falke et al., 1984; Furuya et al., 1984; Fröhlich and Gunn, 1986). Accordingly, the H_2_DIDS-bound AE1 membrane domain adopts an OF conformation with the putative anion-binding site exposed only to the extracellular side.

A variety of alternating-access mechanisms have been described for secondary active transporters, thus far categorized as “rocker-switch,” “rocking-bundle,” and “elevator-type” mechanisms (Drew and Boudker, 2016). The rocker-switch and rocking-bundle models are two variations of the same concept. The former term applies to cases where two protein domains of comparable size move relative to each other (e.g., major facilitator superfamily transporters); because their size is similar, it is assumed that both domains also change their orientation relative to the surrounding membrane as the protein changes from the OF to the inward-facing (IF) state. In contrast, the rocking-bundle concept has been used when the mechanism involves a large unit and a small unit (e.g., a two- or four-helix bundle, like in calcium-cation antiporters or neurotransmitter-sodium symporters, respectively). In this case, it is often assumed that the larger unit is mostly static relative to the membrane because of its greater interface with the lipid bilayer, and therefore, the smaller unit is seen as the mobile element. That this assumption is generally valid is unclear, and thus, the difference between these two types of rocking mechanisms can be quite subtle and somewhat subjective.

Rocking mechanisms, however, differ clearly from elevator-like mechanisms. The distinctive characteristic of the rocking mechanisms is that the binding site remains approximately in place relative to the membrane midplane; to provide access to these sites from either side of the membrane, the moving protein domains reorient around the substrate-binding sites, with one or more of these sites approximately at the pivot point of this motion, in between the two domains (Forrest and Rudnick, 2009). In contrast, the defining feature of elevator-like mechanisms is that the binding site moves perpendicularly to the membrane midplane as the protein cycles between the OF and IF states. As in the rocking mechanisms, two protein domains move relative to each other, but the binding site is not the pivot of this motion, and the site is largely or entirely encompassed by one of the two domains (Vergara-Jaque et al., 2015; Mulligan et al., 2016).

Whether AE1 functions by way of a rocking or elevator-like mechanism (or something entirely different) is not immediately apparent from the existing crystal structure. To gain insights into this mechanism, several studies have compared OF AE1 with structurally related transporters. Specifically, two recent studies have described low-resolution structures of SLC4-type boron transporters, namely a 4.1-Å-resolution crystal structure of occluded-state Bor1 from *Arabidopsis thaliana* (Thurtle-Schmidt and Stroud, 2016), and a 6-Å-resolution structure of IF *Saccharomyces mikatae* Bor1p, obtained with cryoelectron microscopy (Coudray et al., 2017). Confusingly, comparison of these structures with OF AE1 led to divergent conclusions, namely elevator-like in the former study (Thurtle-Schmidt and Stroud, 2016) and rocking bundle in the latter (Coudray et al., 2017). Divergent conclusions have also resulted from comparisons of the AE1 structure with the IF structures of SLC26Dg (Chang and Geertsma, 2017; elevator-like) and the SLC23-family UraA transporter (Reithmeier et al., 2016; rocking bundle). Finally, a combination of intradomain and elevator-like conformational changes was proposed based on comparison of a dimeric UraA structure in an occluded conformation with an IF structure of UapA (Yu et al., 2017). It is likely that one reason why these comparisons have led to such differing interpretations is the low sequence identity between any two of these proteins (<25%), combined with the fact that the structures considered often reflect different functional or oligomerization states. Thus, despite the architectural similarities between these transporters, it is questionable whether these comparisons can be sufficiently precise to infer a mechanism.

**Figure 2.**
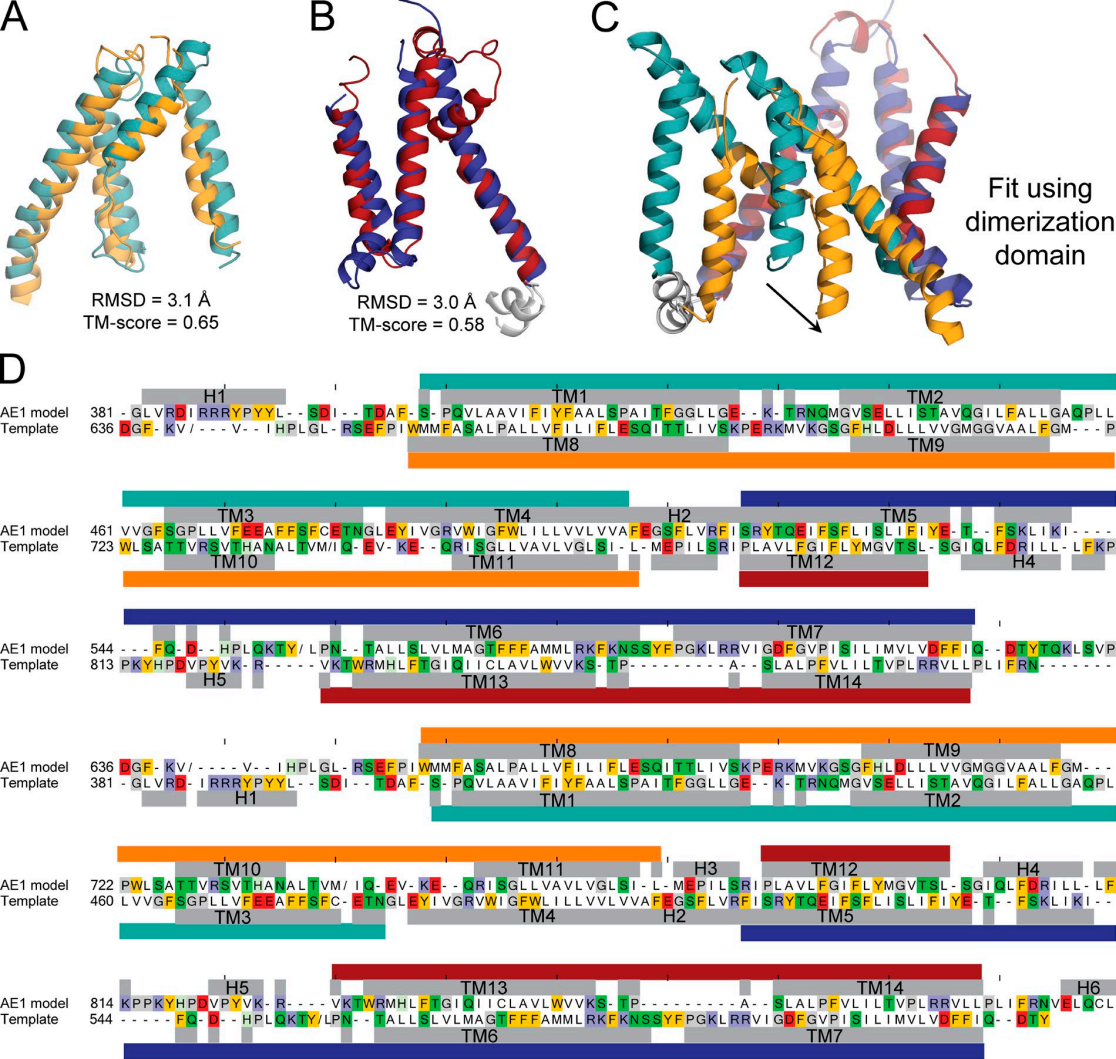
**Asymmetry in the structural repeats forming the membrane domain of AE1, and sequence alignment used to construct a repeat-swapped model. (A and B)** Structural similarity between TM1–TM4 and TM8–TM11 in the transport domain (A) and TM5–TM7 and TM12–TM14 in the dimerization domain (B), compared using TMalign. The corresponding RMSD and TM-score values show that the difference between repeats (Fig. 1 C) is due to the reorientation of two units within each repeat. (A) The transport-domain segments comprise residues 381–508 and 636–775, of which 111 residues are aligned. (B) The dimerization-domain segments comprise residues 509–635 and 776–880, of which 88 residues are aligned. **(C)** Asymmetry of the two structural repeats. **(D)** Sequence alignment between AE1 model and the template, which is the x-ray crystal structure with the order of the repeats exchanged to B then A; the sequence identity is 8.3%. Residues with helical secondary structure according to DSSP are indicated with gray bars and labeled by helix or TM segment. Repeat A is indicated with teal and dark blue bars, and repeat B is indicated with orange and deep red bars (transport and dimerization domains, respectively). “/” indicates a break in the sequence if the segment was not modeled or was not present in the template structure (residues Y553 to L467, V640 to V649, and M741 to I753). Ticks above the model sequence are located every 10 positions for reference. Residues are colored if they are aromatic (gold), basic (indigo), acidic (red), glycine or proline (gray), or polar (green). Residues ELQCL in H6 were modeled using the same element from the crystal structure as a template after applying the transformation matrix that superposes the two repeats.

An alternate strategy to predict the IF conformation of AE1, and thereby the type of transport mechanism used by this protein, is to infer its features from those revealed by a systematic analysis of the known structure. AE1, like most secondary-active transporters (Forrest, 2015), contains two structural repeats with inverted TM topologies (Fig. 1). Repeat “A” comprises TM1 to TM7, and repeat “B” consists of TM8 to TM14. As mentioned, in the folded protein these helices are arranged into two functional domains, each of which includes two fragments of each repeat: TM1–TM4 and TM8–TM11 combine to form the transport domain, whereas TM5–TM7 and TM12–TM14 form the dimerization domain. It is crucial to note that the TM1–TM4 unit is structurally very similar to an inverted version of TM8–TM11; the same applies to TM5–TM7 and TM12–TM14 in the dimerization domain (Arakawa et al., 2015; Fig. 1, C and D; and Fig. 2). However, the orientation of TM1–TM4 relative to TM5–TM7 differs from that of TM8–TM11 relative to TM12–TM14, and therefore, repeats A and B do not have the same structure even though they have identical (but inverted) topologies. This structural difference is in fact crucial, as it is the reason why the putative anion-binding site is exposed to the extracellular space.

It follows from this analysis that a hypothetical model in which repeat A is assumed to adopt the conformation of repeat B, and vice versa, ought to be an IF state. Indeed, this “repeat-swapping” modeling strategy has reliably predicted the global conformational changes of transporters with widely varying structural folds, revealing both rocking-like motions (Forrest et al., 2008; Radestock and Forrest, 2011) and elevator-like motions (Crisman et al., 2009; Vergara-Jaque et al., 2015; Mulligan et al., 2016). We therefore followed this approach to predict the unknown structure of the IF state of the AE1 membrane domain. We then compared this hypothetical model with the OF structure determined experimentally to predict the transport mechanism used by AE1. The OF and IF structures also serve as a framework to interpret the large body of published biochemical, genetic, and transport data, which we review briefly, and suggest new experiments to further evaluate the mechanism of AE1.

## Materials and methods

**Figure 3.**
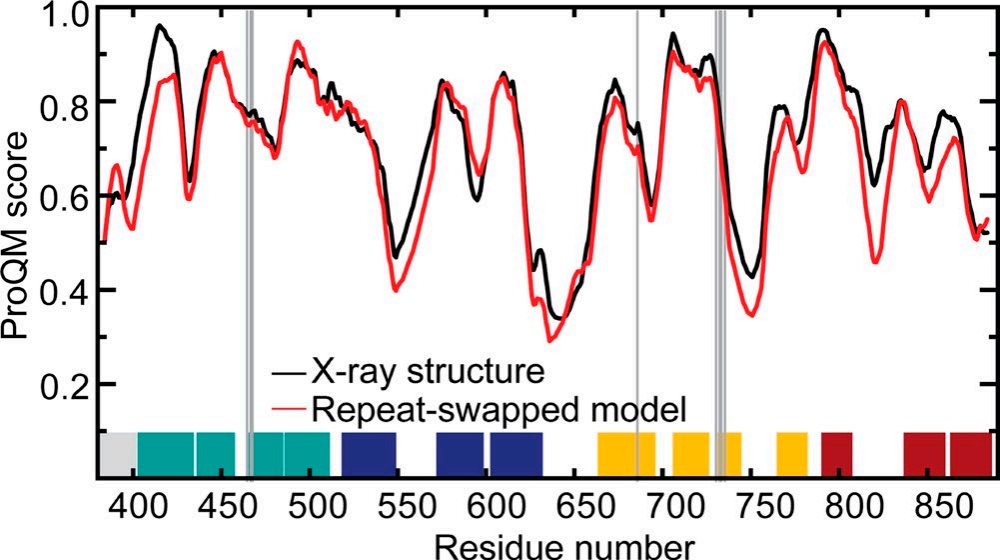
**Structural quality of the AE1 repeat-swapped model compared with that of the x-ray structure.** A five-residue window average of the ProQM score is plotted as a function of residue number. The position of putative binding site residues is indicated by vertical gray lines. The position of TM segments is shown as blocks, colored according to Fig. 1. Data are shown for one protomer of the dimeric model, generated by superposing the dimerization domain onto that of the AE1 crystal structure dimer, whose orientation was determined using the OPM server (Lomize et al., 2006). Clashes at the interface were energy minimized. The global ProQM scores of the minimized crystal structure and model are 0.726 and 0.690, and the MolProbity scores are 2.08 and 1.37, respectively.

An IF model of the membrane domain of AE1 was produced using the repeat-swapping modeling technique described previously (Vergara-Jaque et al., 2015). That is, the template was derived from the OF structure determined by x-ray crystallography (PDB ID 4YZF, chain A; Arakawa et al., 2015; Fig. 2), according to the alignment in Fig. 3. The secondary-structure elements in the OF structure, as assigned by DSSP (Kabsch and Sander, 1983), were assumed to be largely preserved in the IF state and therefore were maintained with helical restraints during the modeling. To allow the IF model to deviate slightly from this assumption, no secondary-structure restraints were imposed on the N- or C-terminal residues in each helix (two residues on either side), on loops connecting subdomains (residues 507–520 including H2 and 774–781 including H3), or on the ends of those loops (four residues on either side). It was also assumed that the IF conformation would preserve the high structural similarity observed in the OF state between TM1–TM4 and TM8–TM11 (transport domain) and between TM5–TM7 and TM12–TM14 (dimerization domain). Therefore, interatomic distance restraints were also imposed for all pairs of Cα atoms within a domain (specifically, residues 382–502 and 664–769 of the transport domain and residues 525–622 and 790–886 of the dimerization domain), provided both atoms are in helical regions and closer than 40 Å in the OF structure. This selection includes over 80% of all residue pairs in each domain. 500 models of the AE1 protomer were thus generated using MODELLER v9.15 (Šali and Overington, 1994). A single model was selected from this set using a set of criteria as described previously (Vergara-Jaque et al., 2015).

A model of the AE1 dimer was then generated by superimposing the dimerization domain in the protomer model onto each of the equivalent domains in the x-ray structure. This IF structure of dimeric AE1 was then energy-minimized with CHARMM version 41b1 (Brooks et al., 1983, 2009) using the CHARMM36 force field (Best et al., 2012) and the GBSW implicit solvent/membrane model (Im et al., 2003). The purpose of this minimization is to remove steric clashes and improve the stereochemistry of the model through small atomic displacements (<1 Å). The position of the dimer in the membrane and its width (36.4 Å) were assumed to be those in the OPM database (Lomize et al., 2006). This procedure comprised several stages in which an increasingly larger set of atoms was included in the energy minimization while others were fixed in space (first hydrogen atoms, then side chains of residues within 10 Å of the protomer interface, then all side chains, then all atoms except Cα atoms, and finally all atoms). Each stage included 500 steps of steepest descent and 1,000 steps of ABNR minimization. The root-mean-square deviation (RMSD) of the backbone as a result of these energy minimizations was 1.1 Å.

### Online supplemental material

Video 1 shows the predicted conformational change obtained by morphing the OF structure into the IF model. Data S1, included as a TXT file, shows the IF model in Protein Data Bank format.

## Results

The asymmetry observed in the H_2_DIDS-bound crystal structure of AE1 (i.e., that the structure is an OF conformation) stems from the asymmetry of the two inverted-topology seven-helix repeats (Figs. 1 and 2). Specifically, these repeats differ in the relative orientation of the four helices contributing to the transport domain and the three helices contributing to the dimerization domain (individually, these four- and three-helix units are nearly identical in both repeats). When the two repeats intertwine, this difference translates into a greater separation between the transport and dimerization domains on the extracellular side (i.e., an OF state; Figs. 1 and 2). It follows from this reasoning that a hypothetical conformation in which each repeat adopts the structure of the other should represent an IF conformation. However, whether the experimental structure and such a hypothetical model could interconvert via a physically plausible mechanism, and what type of mechanism that might be, are not self-evident.

**Figure 4.**
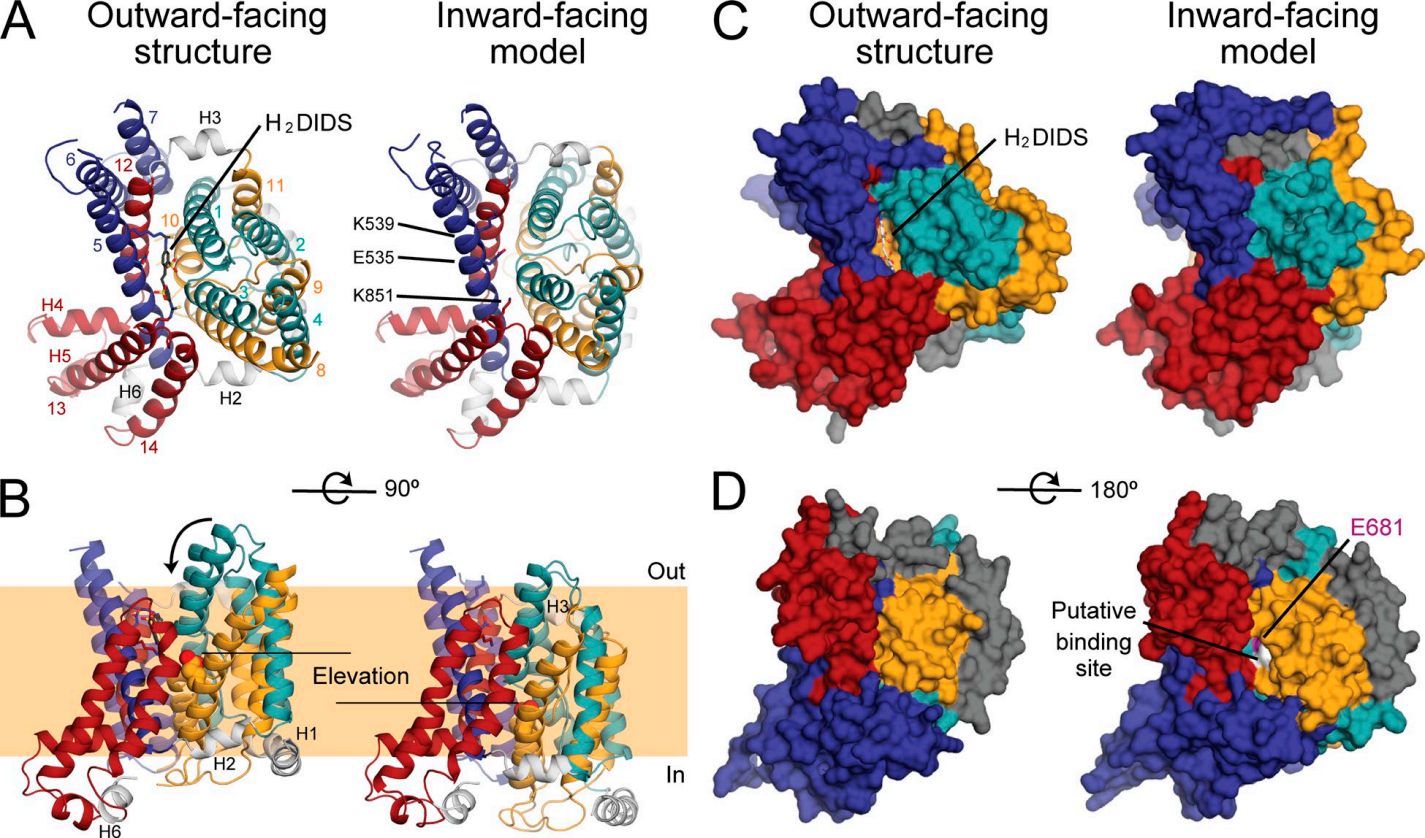
**Comparison of a repeat-swap model of AE1 with the OF H_2_DIDS-bound x-ray crystal structure predicts an elevator-like conformational change, where the transporter domain (cyan and yellow) rotates and moves inwards, relative to the scaffold domain (red and blue). (A–D)** Individual protomers are viewed and helices are colored according to Fig. 1. The structures are viewed from the extracellular side (A and C), the plane of the membrane, highlighting the inward movement of the transport domain and the putative substrate-binding site encompassed therein (B), or the cytoplasmic side (D). The protein is shown as cartoon helices (A and B) or surface (C), with H_2_DIDS and residues K539, E535, and K851, which line the pathway in the OF structure, shown as sticks. The E681 side chain, which is thought to be at the substrate binding site, is shown as spheres. This extracellular pathway is closed in the repeat-swapped model (C). In contrast, a new pathway opens up from the cytoplasmic side (D) that leads to residues assumed to form the anion-binding site (white). The model predicts a two-domain motion, where the teal and orange helices are displaced by ∼11 Å and rotated by ∼17°. This movement requires the linker helices H2 and H3 (gray cartoon helices) to change their tilt angle relative to the membrane surface.

We therefore constructed a model of the IF state of the membrane domain of AE1 using the so-called repeat-swap homology modeling method, in which the conformations of the two repeats are exchanged. In the H_2_DIDS-bound structure, the presumed substrate-binding site is located between the ends of the helical portions of TM3 and TM10 in the transport domain and is accessible to the extracellular side of the membrane (Arakawa et al., 2015; Fig. 4). In stark contrast, in the repeat-swapped model, the putative anion-binding site is exposed to the cytoplasm and not to the extracellular side. The observation that the repeat-swapped model represents an IF state (Fig. 4, C and D) validates the underlying premise of this modeling approach, namely that the asymmetry in the global structure of the transporter results from the asymmetry in the internal structure of the two topological repeats. Based on a linear interpolation (Video 1), it appears that the interconversion between the experimental OF structure and the IF model would not be sterically hindered and also perfectly compatible with two additional assumptions in our modeling procedure; namely, that this transition preserves the secondary structure of the protein as well as the individual tertiary structure of the transport and dimerization domains. Finally, this transition does not require any changes at the dimerization interface, which can therefore be thought as stationary. In contrast, the transport domain containing the anion-binding site (TM1/TM8 and TM3/TM10) is predicted to translate by ∼11 Å (equivalent to approximately two turns of a helix) while simultaneously rotating ∼17° around an axis perpendicular to the membrane and centered away from the dimer interface (Fig. 4 B and Video 1). The component of that translation along the membrane normal is ∼8 Å, suggesting that AE1 uses an elevator-like mechanism to alternatively expose the anion-binding site to either side of the membrane.

In both conformations of AE1, the transport domain helices TM1/TM8 and TM3/TM10 are pitched ∼30° from the membrane normal. The dimerization domain helices TM5 and TM12 at the domain–domain interface are also pitched ∼30° from the membrane normal, but in the opposite direction. As a result, in the transition from the OF to IF state, TM1/TM8 and TM3/10 in the transport domain would move across, rather than along, the dimerization domain helices TM5 and TM12. In the OF state, the anion-binding pocket in the transport domain is adjacent to the middle of TM5 in the dimerization domain. The elevator-like motion of the transport domain predicts that the anion-binding pocket would move to a position in the IF state that is nearly opposite to (and toward the cytoplasmic end of) TM12 in the dimerization domain.

**Figure 5.**
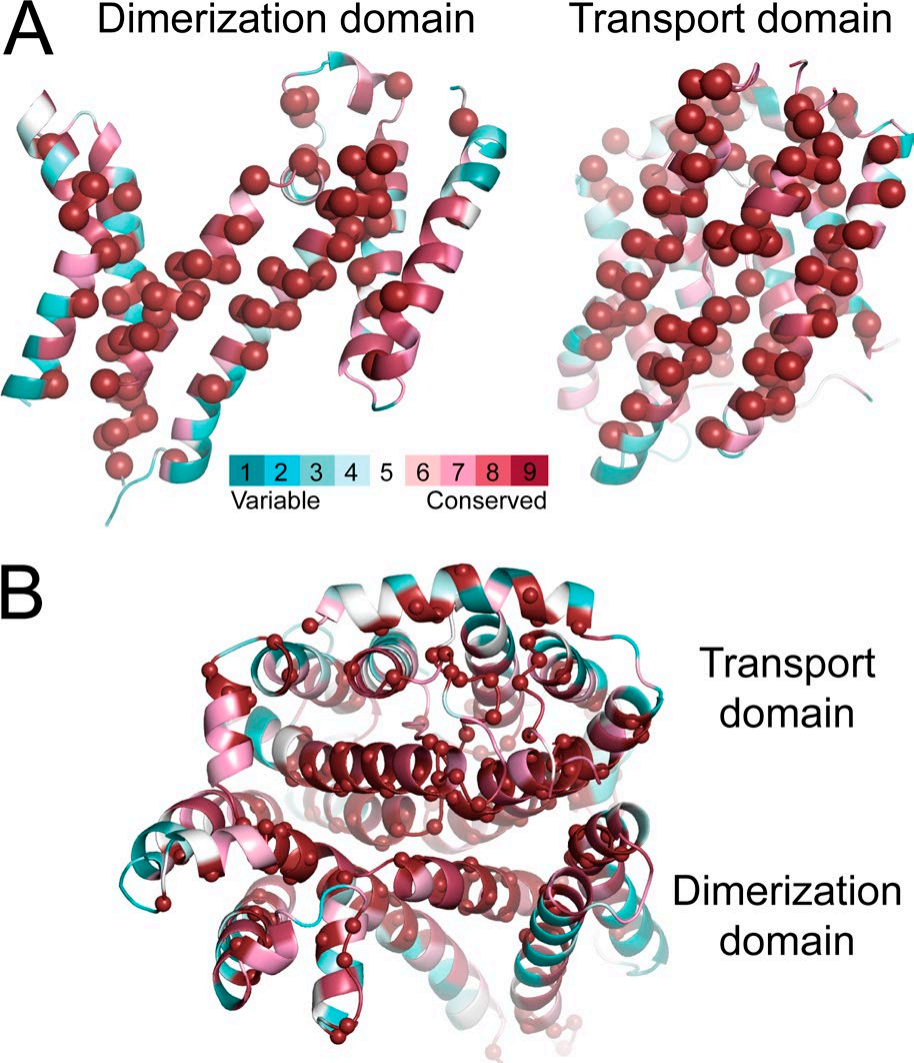
**Conservation of the residues at the interface between the transport and dimerization domains of AE1. (A and B)** The x-ray structure (A), showing each domain as viewed from the other domain, and the repeat-swapped model (B), viewed from the intracellular side of the membrane. Residues are colored according to conservation from blue to burgundy, and those with the maximum conservation level are also indicated by spheres. Conservation was computed with the ConSurf webserver (http://consurf.tau.ac.il) using a PSI-BLAST search on January 4, 2016.

In both states, the substrate anion must diffuse from either the extracellular or intracellular medium to the substrate-binding site along an access pathway lined by the transport and dimerization domains. The residues forming those pathways, and the contacts between the two domains, are highly conserved (Fig. 5). In the OF state, the pathway is lined by TM1 and TM3 from the transport domain and TM5 and TM13 from the dimerization domain; H_2_DIDS binds in this pathway (Fig. 4 A). In the predicted IF state, the access pathway is lined by the symmetry-equivalent helices, TM8 and TM10 in the transport domain and TM7 and TM12 in the dimerization domain.

To accommodate the rearrangements between the two domains in the transition from the OF to IF state, the model predicts changes in the orientation of the two short helices (H2 and H3) that connect the two domains (Fig. 4 B). The axis of H2, on the cytoplasmic surface connecting TM4 in the transport domain and TM5 in the dimerization domain, tilts ∼20° to allow TM4 to move inward by ∼8 Å relative to TM5. The axis of H3, which is on the extracellular surface connecting TM11 in the transport domain and TM12 in the dimerization domain, is nearly parallel to the membrane in the OF state and is predicted to tilt ∼30° in the translocation event, allowing TM11 to move inward relative to TM12 (Fig. 4 A). Only the short connector helices, not the TM helices, change tilt angle by >10° during the translocation event.

In summary, the repeat-swapped model of AE1 predicts an elevator-type transport mechanism rather than a mechanism involving rocking of helix bundles around a stationary substrate-binding site. Instead, in this elevator-like mechanism, it is the dimerization domain that remains stationary, whereas the transport domain, which encloses the substrate, moves vertically relative to the membrane plane and rotates around the membrane perpendicular. Although these motions are apparent when the OF crystal structure is compared with our IF model, it is important to note that our prediction might underestimate the actual range of these motions. It is quite possible that the H_2_DIDS-bound structure of AE1 is less outward-open than the actual unbound state. The monoclonal antibody fragment used to scaffold the AE1 crystal might also restrict the degree to which the anion-binding site is exposed to the extracellular space because this Fab fragment specifically recognizes the transport domain and docks onto that side of the protein. A repeat-swapped IF model derived from a more outward-open structure would reveal an even larger vertical displacement of the transport domain, providing further support to the proposed elevator-like mechanism.

## Discussion

From comparison of the crystal structure of OF AE1 and the IF model we constructed, it can be clearly inferred that this antiporter features an elevator-like mechanism. It is important to note that the strategy used to develop the IF model does not presuppose any type of mechanism; indeed, based on the same procedure, it has been concluded that other transporters feature a rocking-type mechanism (Forrest et al., 2008; Radestock and Forrest, 2011). Although this IF model is hypothetical at this point, we can begin to examine whether an elevator-like mechanism is compatible with earlier biochemical, structure–function, and genetics studies of AE1 (Passow, 1986; Tanner, 1993; Hamasaki and Okubo, 1996; Wrong et al., 2002; Reithmeier et al., 2016).

### Intermolecular and intramolecular cross-linking studies

The contacts between protomers in the crystal structure of AE1 are primarily in TM5, TM6, the extracellular loop between them, and the TM6–TM7 cytoplasmic loop (Arakawa et al., 2015; Fig. 1 A). By construction, these contacts are maintained in the repeat-swapped IF model. That is, we hypothesize that the dimerization domains remain largely stationary during transport. This element of our hypothesis is consistent with the evidence that the mechanism of one protomer is independent from the other (Jennings, 1984; Reithmeier et al., 2016) and with the finding that the protomers can be cross-linked using BS^3^ at sites in the TM5–TM6 loop (K551 on one subunit and K562 on the other) without inhibiting transport (Jennings et al., 1985; Jennings and Nicknish, 1985; Fig. 5 A). A stationary oligomerization domain seems to be a shared feature of transporters exhibiting elevator-like mechanisms (Erkens et al., 2013; Mulligan et al., 2016; Ruan et al., 2017).

Although no structure of full-length AE1 is yet available, a recent cross-linking study suggested that salt-bridge interactions can form between the cytoplasmic domain and both the dimerization and transport subdomains (Rivera-Santiago et al., 2017). These data might appear to be incompatible with the kind of conformational changes we propose. However, owing to the irreversibility of the cross-linking reaction, interactions that form only transiently or at different stages of the transport cycle would appear as permanent and concurrent in this methodology. Thus, without complementary assays to ascertain the functionality of the cross-linked protein, these data do not confirm or rule out an elevator-like or a rocking-type mechanism.

### Alternating accessibility of E681

In addition to the physiological substrates Cl^−^ and HCO_3_^−^, AE1 catalyzes SO_4_^2−^ transport as SO_4_^2−^:H^+^ cotransport (Jennings, 1976), with E681 in TM8 as the apparent proton-binding site (Jennings and Smith, 1992; Chernova et al., 1997). Consistent with this role, in the OF crystal structure of AE1 (Arakawa et al., 2015), E681 is close to the putative anion-binding site and accessible to the extracellular medium (if H_2_DIDS is removed). Chemical modification of E681, however, also strongly affects the intracellular pH dependence of SO_4_^2−^ translocation, indicating that E681 traverses the permeability barrier during transport (Jennings and Al-Rhaiyel, 1988). Indeed, in our IF model, E681 is accessible from the cytoplasmic side of the membrane (Fig. 4 D), lending support to the proposed elevator-like mechanism.

### Cysteine-scanning mutagenesis

**Figure 6.**
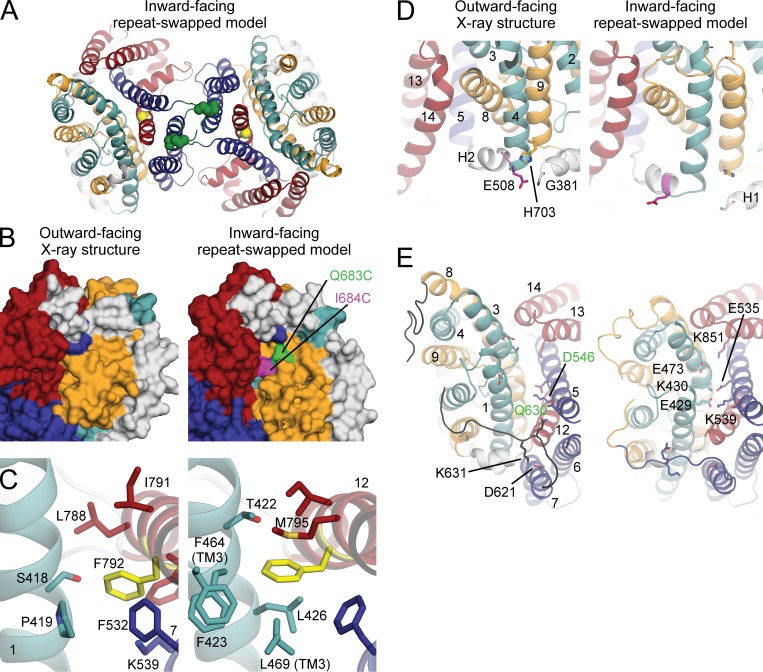
**Consistency between the proposed elevator-like mechanism and previous experimental findings. (A)** In the proposed elevator mechanism based on the repeat-swapped model (cartoon helices), the transport domain (teal and orange) is predicted to move relative to the dimerization domain (dark blue and dark red). Dimerization by cross-linking residues K551 (spheres) and K562 (not in known structure or model) from the TM5–TM6 loop (dark green) does not inhibit anion transport, consistent with their predicted location. Residue G796 in TM12 is in the domain interface (yellow sphere). **(B)** Biotinylation demonstrates accessibility of I684C (magenta) and, weakly, of Q683C (green) in TM8. These residues are only visible in the predicted cytoplasmic pathway of the IF model of AE1 (right) and not in the OF structure (left). **(C)** F792 (yellow sticks) in the dimerization domain forms more hydrophobic contacts with TM1 and TM3 of the transport domain in the model (right) than in the structure (left). Residues with C atoms <4.3 Å from an atom of F792 are shown as sticks. Helices are shown as cartoon helices (except TM3, for clarity) and labeled in bold. **(D)** E508 (magenta) at the cytoplasmic end of TM4 form contacts with G381 (white) and H703 in the OF structure (left) that may be lost in the IF conformation (right) as the distance between TM4 and TM5 increases. **(E)** Several ionizable residues on the extracellular surface (sticks) are predicted to form salt bridges only in the IF (right) or OF conformation (left). Three of these putative interactions line the extracellular pathway, bridging the transport and dimerization domains. The papain cleavage site (Q630) and its interaction partner in TM5 (D546) are also shown (green labels); the cleavage sites Q550 and Q564 are in the unresolved loop between TM5 and TM6. The 40-residue-long loop connecting TM7 and TM8 is colored black.

Cysteine-accessibility studies revealed that biotin had limited access to residues 664–682 in TM8 but that the accessibility increased slightly at Q683 and more abruptly at I684 (Tang et al., 1998). The OF crystal structure does not explain this pattern, because I684 is not accessible from either side of the membrane (Fig. 6 B). However, under the labeling conditions used by Tang et al. (1998), AE1 also visits the IF state, and indeed, in our predicted model, Q683 and I684 become accessible from the intracellular medium (Fig. 6 B). The elevator mechanism therefore provides a straightforward explanation of the biotinylation pattern of residues in TM8.

In the loop preceding TM8, cysteine substitutions of W648, I650, P652, L655, and F659 strongly inhibit anion exchange (Tang et al., 1998). This ∼40-residue extracellular loop links the dimerization and transport domains, and in the OF structure, several of these residues form hydrophobic contacts with TM4 and TM9 in the transport domain. As an unstructured loop with minimal contacts, the reliability of this region in the repeat-swapped model is likely to be low (Fig. 3). Nevertheless, the predicted mechanism would result in significant rearrangements in this loop, which would be consistent with the inhibitory effect of modifications of this region.

### Alanine-scanning mutagenesis

Transport by AE1 is inhibited by substituting alanine for G463 or S465 (Bonar et al., 2013), which can be explained by their location near the proposed anion-binding site at the N terminus of TM3. However, alanine substitution is also inhibitory at F792 on TM12, at the interface between the dimerization and transport domains (Fig. 6 A). This interface would reconfigure during the predicted transport cycle and influence the mechanism. The repeat-swapped model predicts at least four hydrophobic side chains in the vicinity of F792, unlike in the OF structure (Fig. 5 C). Therefore, we posit that in F792A, the IF conformation is destabilized, possibly trapping the transporter in the OF state.

Replacement by either alanine or threonine at F878, between TM14 and the short helix H6, also leads to inhibition, suggesting that a large side chain is required to retain packing with TM13 in this region. However, all contacts formed by F878 are maintained in both the OF structure and the IF model, and therefore, this mutation does not specifically confirm the elevator-like mechanism. Finally, alanine substitution at E508 accelerates transport by ∼50%; serine, aspartate, and lysine substitutions result in an even faster rate (Bonar et al., 2013). E508 is in the connector between TM4 and H2 and faces H703 of TM9, the N-terminal residues of H1, and the lipid head-group region (Fig. 6 D). In the predicted elevator mechanism, the angle between H2 and TM4 changes by ∼13° in the transition from the OF to IF state, and the interactions of E508 with H1 and TM9 and/or with the lipids are predicted to be lost. However, residues presumed to be near E508 (Rivera-Santiago et al., 2017) and immediately N-terminal to H1 are not present in the crystal structure and consequently also not in the repeat-swapped model. Thus, although the effects of mutations on transport in the hinge region between TM4 and H2 are consistent with an elevator mechanism, other factors may also be at play.

### Cleavage of extracellular loops with papain

Extracellular papain cleaves the TM5–TM6 loop at residues Q550 and Q564 without any detectable effect on anion transport (Jennings et al., 1984), consistent with the proposal that the internal structure of the dimerization domain is largely invariant (Fig. 6 E). In contrast, a pronounced effect on anion transport results from cleavage at Q630, which is located in the TM7–TM8 loop linking the dimerization and transport domains (Jennings et al., 1984). This observation is consistent with the hypothesis that the TM7–TM8 loop undergoes a substantial conformational change due to the displacement of the transport domain (Fig. 6 E).

It is worth examining further the specific functional effect of this cleavage. AE1 activity is inhibited by ∼90% if transport is measured as exchange flux with symmetric substrate concentrations (Jennings and Passow, 1979; Jennings and Adams, 1981). Surprisingly, though, if transport is measured under influx-limited conditions (either a slow external exchange partner like SO_4_^2−^ or low extracellular Cl^−^ concentration), cleavage of the TM7–TM8 loop accelerates transport (Jennings and Adams, 1981). The effect of papain cleavage at Q630 could be interpreted as lowering the rate of outward anion translocation while increasing the rate of inward translocation. That is, disruption of the TM7–TM8 loop would either stabilize the IF state or destabilize the OF conformation. Comparison of the crystal structure and the IF model suggests the former possibility is the more likely. In the structure, the TM7–TM8 loop forms contacts with both the dimerization and transport domains, which appear to be compatible with cleavage at Q630. Although modeling of loop regions is generally not very accurate (Fig. 3), in the IF model it appears clear that the TM7–TM8 loop does not simultaneously maintain these contacts with both domains. Cleavage at Q630 could restore these interactions, which would be then preserved throughout the cycle, effectively shifting the conformational equilibrium of the transporter toward the IF state. Notably, the effect of cleavage of the AE1 TM7–TM8 loop is qualitatively similar to that of cleavage of loop 3L4 in an archaeal glutamate transporter homologue (Mulligan and Mindell, 2013). That trimeric transporter is known to use an elevator-type mechanism and loop 3L4 connects the trimerization and transport domains. These observations support the predicted elevator-like mechanism also in AE1 while providing a rationale for the long-unexplained effect of papain cleavage.

### Deletion in Southeast Asian ovalocytes

Southeast Asian ovalocytosis (SAO) results from the deletion of nine amino acid residues (A400–A408) near the N terminus of the AE1 membrane domain (Liu et al., 1990; Jarolim et al., 1991). SAO-AE1 inserts in the membrane (Schofield et al., 1992b; Groves et al., 1993) and forms dimers (Jennings and Gosselink, 1995) but does not transport anions or bind stilbenedisulfonates (Schofield et al., 1992a).

**Figure 7.**
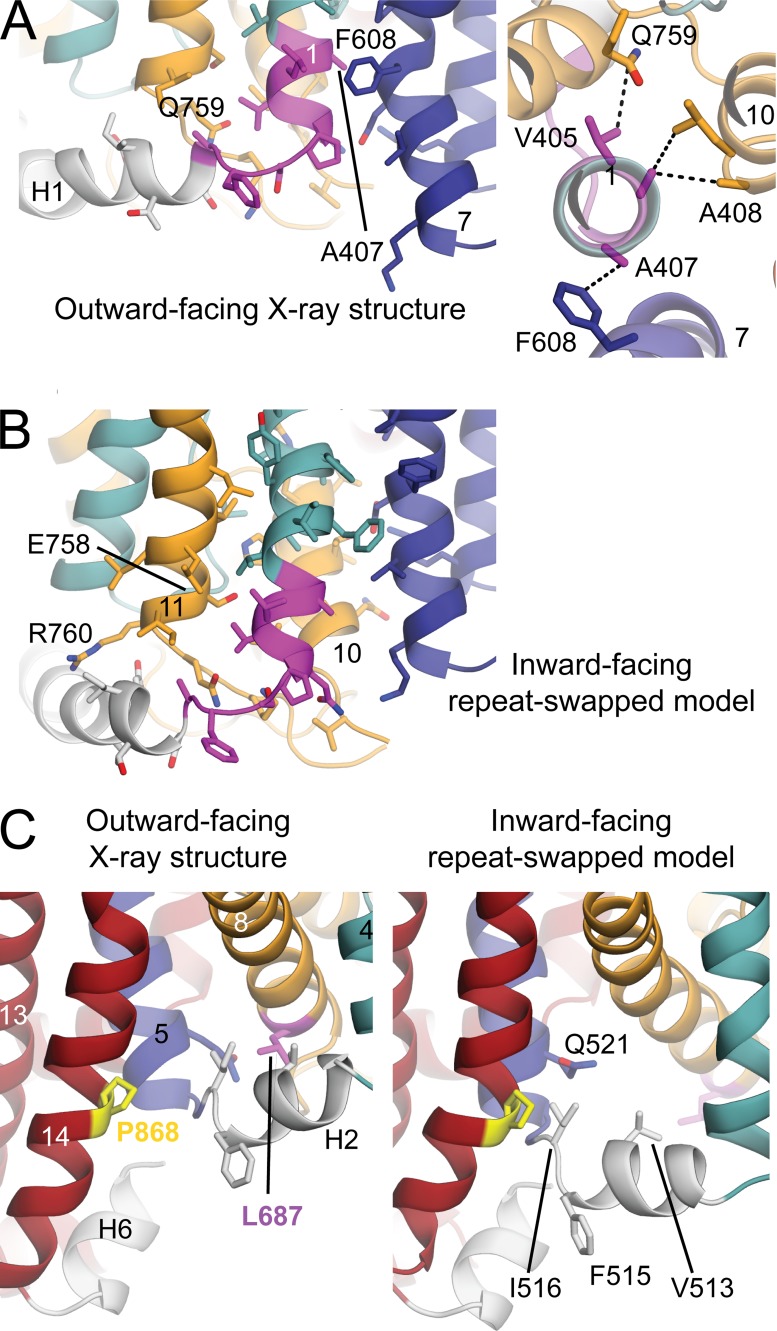
**Consistency between the proposed elevator-like mechanism and known disease-related mutations. (A and B)** SAO deletion of residues 400–409 (magenta) in H1/TM1 would cause loss of interactions to TM7 and TM10 found in the OF state (A) but not present in the predicted IF state (B). (A) The AE1 structure (PDB ID 4YZF) is viewed along the plane of the membrane, with the cytoplasm at the bottom (left), or from the extracellular side (right). (B) The repeat-swapped model of AE1 is viewed along the plane of the membrane. **(C)** Mutations at positions interacting with H2. P868 (yellow) of TM14 is mutated to P868L in the high-transport (HT) variant, and L687 (magenta) of TM8 is mutated to L687P in hereditary stomatocytosis (HSt). P868 is predicted to form closer contacts with L516 in the H2 linker in the IF repeat-swapped model due to the reorientation of the transport domain relative to the dimerization domain. In contrast, L687 forms contacts with I516 and V513 of H2 and Q521 of TM5 in the OF conformation that are predicted to be lost in the IF state.

In the OF crystal structure, residues 400–408 comprise the C terminus of cytoplasmic helix H1 and the first six residues of TM1 (Arakawa et al., 2015; Fig. 7 A). If the SAO-AE1 structure globally resembles that of normal AE1, then SAO deletion would place D399 of helix H1 adjacent to V409 at the N terminus of TM1, disrupting native interactions within the transport domain between TM1 and, for example, TM10. Probably more critically, the SAO deletion would disrupt interactions around the cytoplasmic ends of TM1 and TM7 (A407 and F608; Fig. 7 A); i.e., between the transport domain and the dimerization domain. These segments form close contacts in the OF state structure, but not in the predicted IF state (Fig. 7 B), due to the movement of the transport domain. We therefore speculate that the SAO deletion destabilizes the OF structure (consistent with the fact that the SAO subunits do not bind H_2_DIDS), leading to the observed loss of transport function. Thus, the SAO mutation appears to be consistent with an elevator-like mechanism.

### The AE1 HT mutation

The AE1-HT human genetic variant carries the mutation P868L, which causes the V_max_ for anion exchange to be two- to threefold higher than that of the wild-type transporter (Bruce et al., 1993). In the crystal structure of AE1, P868 causes a bend in TM14 on the surface of the dimerization domain (Fig. 7 C). Like the rest of this domain, TM14 moves little in the predicted translocation event. However, nearby cytoplasmic helix H2, which connects the transport and dimerization domains, is predicted to undergo a major reorientation, which specifically appears to change its packing against TM14 (e.g., F515 and I516). Thus, the two possible consequences of the P868L mutation are (a) TM14 straightens in the absence of a helix-breaking proline, somehow altering the compact architecture of the dimerization domain and its relationship with the transport domain or the membrane; or (b) the interactions between TM14 and H2 are altered at one or other stage in the cycle because of the larger side chain. Either possibility would be consistent with the proposed elevator-like mechanism.

### Other naturally occurring point mutations

Several mutations in the human AE1 gene cause erythrocyte abnormalities (Table 1), such as spherostomatocytosis and spherocytosis and/or distal renal tubular acidosis (dRTA; Maillet et al., 1995; Bruce et al., 1997, 2000, 2005; Reithmeier et al., 2016). Two of these mutations, L687P and G796R, are associated with a significant decrease in transport activity. L687P in TM8 may destabilize the OF conformation by altering contacts with residues in H2 (Fig. 7 C), whereas G796R in TM12 would place a large charged residue in the domain interface and likely impede the elevator mechanism (Fig. 6 A). Thus, both L687P and G796R appear to be consistent with the elevator-like mechanism.

**Table 1.** Naturally occurring point mutations causing hereditary stomatocytosis or spherocytosis and severe loss of anion transport in red cells and or AE1 expressed in *Xenopus* oocytes

**Mutation (reference)**	**Location in the OF state and movement in the elevator mechanism**	**Elevator mechanism: Support, refute, or neutral?**
HS C479W (Chu et al., 2010) found in compound heterozygote with dRTA mutation G701D	C479 is near extracellular end of TM3, facing TM4 and TM8. It moves inward with the transport domain in the translocation event.	Neutral. Tryptophan substitution could disrupt packing near the ends of TM4 and TM3, which could propagate to the binding site, but there are no major conformational changes here in the elevator mechanism.
HSt R730C, S731P (Bruce et al., 2005; Guizouarn et al., 2007; Stewart et al., 2011)	At the N-terminal end of TM10, near the substrate-binding site and expected to move with the substrate and the rest of the transport domain.	Neutral. Mutations at these sites would be expected to affect transport for almost any mechanism.
HSt H734R, S762R (Bruce et al., 2005)	These two residues are close to each other in the OF state.	Neutral. Arginine substitution is expected to disrupt packing in the transport domain.
HS D705Y (Bruce et al., 2005; Guizouarn et al., 2007; Barneaud-Rocca et al., 2013),	Near the cytoplasmic end of transport TM9, facing TM2 and nonhelical residues leading to TM3.	Neutral. Tyrosine substitution would interfere with packing within the transport domain.
HSt L687P (Bruce et al., 2005; Guizouarn et al., 2007; Stewart et al., 2011)	Cytoplasmic end of TM8 (transport), near Q521 of TM5 (dimerization) and V513, I516 of short helix H2. L687 moves several angstroms away from Q521, V513, and I516 in the translocation event.	Support. Proline for leucine would cause a bend near the end of TM8. Moreover, the smaller side chain would also likely have weaker interactions with H2, therefore destabilizing the OF state.
HSt G796R (Iolascon et al., 2009)	In TM12 (dimerization) close to the transport-dimerization interface. Residue does not move in translocation, but substrate-binding site moves directly past G796.	Support. Arginine replacement of glycine in this position would be expected to impede elevator motion.

HS, hereditary spherocytosis; HSt, hereditary stomatocytosis.

Other mutations in the membrane domain that cause erythrocyte abnormalities have milder effects on the rate of anion exchange (Table 1), including E758K and R760Q (Ellory et al., 2009; Stewart et al., 2010). E758 and R760 are located at the cytoplasmic end of TM11, in the transport domain (Fig. 7 B). In the elevator mechanism, E758 and R760 move with the rest of the transport domain, without major changes in local conformation. Thus, the fact that these mutations do not strongly inhibit anion transport is consistent with the predicted elevator model. We speculate that the disease-causing effect of these mutations relates instead to their contacts with H1 and/or the cytoplasmic domain preceding H1 that anchors AE1 to the membrane cytoskeleton and thereby controls erythrocyte morphology.

Other human AE1 mutations that cause dRTA (Quilty et al., 2002; Wrong et al., 2002; Devonald et al., 2003; Batlle and Haque, 2012), preserve, or have only minor effects on, transport function (R589H, G701D, S613F, Δ850, A858D, and G901Stop; Bruce et al., 1997, 2000; Jarolim et al., 1998; Toye et al., 2002; Shmukler et al., 2010). These sites are located within helices TM6, TM7, TM9, TM13, and TM14, where modifications may cause local perturbations in structure that could affect trafficking. However, none of these sites are predicted to undergo major changes in interactions in the proposed mechanism, which would therefore explain why the transport function of these mutants is preserved.

### Electrogenicity of the anion translocation events

As mentioned, the Cl^−^/ HCO_3_^−^ exchange cycle of AE1 is electroneutral. The Cl^−^/SO_4_^2−^ exchange cycle is also electroneutral, as it involves cotransport of H^+^ (Jennings, 1976) carried by E681 (Jennings and Smith, 1992). Each of the individual anion-translocation events is also very close to being electroneutral (Grygorczyk et al., 1987; Jennings et al., 1990). This finding has two implications: first, that the protein counterbalances the electrostatic charge of the bound anion; and second, that in the OF and IF conformational states in which the anion is loaded and released, the binding site is in a location where the transmembrane electrostatic potential is similar to the bulk value.

The neutralization of the anion is likely effected by R730, the N-terminal dipoles of TM3 and TM10, and E681. The proposed mechanism offers an intuitive explanation of the experimental results in that this neutral binding site literally moves between the extracellular and intracellular compartments, likely traversing most of the TM electric field. In contrast, a mechanism whereby the binding site is stationary and deep into the membrane would likely require the anion to traverse a nonnegligible portion of the electric field before becoming charge neutralized. Although a rocking mechanism cannot be categorically ruled out based on this qualitative argument alone, the elevator-like mechanism seems more conducive to an electroneutral process.

### Modulation of function by extracellular pH

SO_4_^2−^ efflux is strongly inhibited by low extracellular pH, even when E681 is neutralized by mutation (Chernova et al., 2008), because of unidentified protonatable groups. There are several carboxyl groups and one histidine residue that could be titratable from the extracellular medium in both OF and IF AE1, a few of which are predicted to be in salt-bridging distance with lysine residues in the opposing domain in one of the two states. Specifically, the repeat-swapped model predicts close proximity of E535 (TM5) and K430 (TM1), E429 (TM1) and K539 (TM5), and E473 (TM3) and K851 (TM13), whereas the interaction between D621 (TM7) and K631 (TM7–TM8 loop) is predicted to be lost (Fig. 6 E). It is plausible that this network of interactions is sensitive to protonation at low extracellular pH, which would presumably decrease the stability of the IF state. Thus, the repeat-swapped model appears to provide a rationale for the observed effects of extracellular pH.

### Conclusions and future directions

Comparison of the OF crystal structure of human AE1 with a repeat-swapped model of the IF state strongly suggests that this important protein utilizes an elevator-like mechanism of transport, akin to that observed in, for example, excitatory amino acid transporters and their homologues. This structural comparison is thus inconsistent with a rocking-type mechanism, akin to that observed in, for example, major facilitator superfamily transporters or neurotransmitter-sodium symporters. The hypothesized elevator-like mechanism provides a specific, structural rationale for a wide range of functional observations, such as the effect of deleterious and nondeleterious mutations and cleavages of the protein and the change in accessibility of specific residues. We note, however, that these experimental data might also be consistent with other transport mechanisms. Nevertheless, in the absence of a plausible model of the IF state differing from that reported here, we cannot evaluate this possibility. Such a model is not available, because an alternative, unbiased modeling strategy based on clearly defined premises, such as that used here, is not apparent. For example, to our knowledge, no suitable template structure is available for conventional homology modeling. Thus, until such time as an experimental structure of IF AE1 or a closely related homologue can be determined, it is hoped that the model proposed here will stimulate further work, both experimental and computational, on AE1 and related proteins.

Experimentally, it may be possible to test the predicted elevator-like mechanism using cross-linking experiments with selected cysteine substitutions, following an approach similar to that used to study other elevator-like transporters (Crisman et al., 2009; Mulligan et al., 2016), or using linkers of different lengths (Sun and Kaback, 1997). Other possible tests of the elevator mechanism include chemical labeling experiments with substituted cysteines (Tang et al., 1998, 1999; Fujinaga et al., 1999) while using Cl^−^ gradients to overpopulate either the IF or OF state. Regions of interest include TM1, TM3, TM8, and the short helix H3. The proposed structure of the IF state could also be tested using biochemical approaches (labeling and proteolysis) in human red blood cells. Further analysis of the F792A mutation at the interface between the dimerization and transport domains is also likely to be revealing.

Finally, it is of interest to ask whether the elevator model also applies to the Na^+^-HCO_3_^−^ cotransporters in the SLC4 family (Romero et al., 2004, 2013). Though structurally similar, a fundamental difference between cotransporters and exchangers is that cotransporters transition between the OF and IF states only if all substrates or no substrate is bound. If the elevator model also applies to SLC4 cotransporters, then a comparison of SLC4 cotransporters and exchangers could produce new insights into the fundamental nature of coupled transport (i.e., the rules governing what substrates must be bound for translocation to take place).

## Supplementary Material

Supplemental Materials (PDF)

Video 1

Data S1
