# Supplementary material for: Asymmetry of inverted-topology repeats in the AE1 anion exchanger suggests an elevator-like mechanism
Source: J Gen Physiol. 2017 Dec 4;149(12):1149–64. doi: 10.1085/jgp.201711836 (PMC5715908; doi:10.1085/jgp.201711836)
Supplement: Supplemental Materials (PDF) [file JGP_201711836_sm.pdf]

The Journal of General Physiology

SUPPLEMENTAL MATERIAL

Ficici et al., https://doi.org/10.1085/jgp.201711836

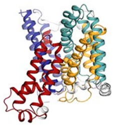

Video 1. **Predicted elevator-like conformational change of anion exchanger AE1, obtained by morphing the OF structure into the IF model using the PyMOL Molecular Graphics System (v1.8.6; Schrödinger, LLC).** The single promoter is viewed along the plane of the membrane, with the dimerization domain on the left. Residues near the binding site are shown as spheres. Helices are shown as ribbons and colored by repeat segment (according to Fig. 1).

Data S1, included as a TXT file, is the structural model of AE1 in an IF conformation, constructed by exchanging the conformations of the repeats in the OF structure.
